# 

**Published:** 1884-03

**Authors:** 


					﻿Vol. V.
MARCH, 1884.
No. 3.
THE
liidmmiPnflilifliiti:
> Monthly Journal
DEVOTED TO
DENTAL AND ORAL SCIENCE.
W. C. BARRETT, M. D., D. D. S-,
ATOTTO ~R. _
The New Yore Dental Journal Association,
PUBLISHERS,
No. 35 West JLGth. Street, New Yor'c.
$2.50 Per Annum in Advance, .... Single Copies, 25 Cents.
Rntprpd nt th** Past-Officp Ruffnln \r V. as	maHpr
				

